# Spontaneous Healing of *Mycobacterium ulcerans* Lesions in the Guinea Pig Model

**DOI:** 10.1371/journal.pntd.0004265

**Published:** 2015-12-01

**Authors:** Rita Silva-Gomes, Elly Marcq, Gabriela Trigo, Carine M. Gonçalves, Adhemar Longatto-Filho, António G. Castro, Jorge Pedrosa, Alexandra G. Fraga

**Affiliations:** 1 Life and Health Sciences Research Institute (ICVS), School of Health Sciences, University of Minho, Braga, Portugal; 2 ICVS/3B’s—PT Government Associate Laboratory, Braga/Guimarães, Portugal; 3 Mycobacteriology Unit, Department of Biomedical Sciences, Institute of Tropical Medicine, Antwerp, Belgium; 4 Institute for Biotechnology and Bioengineering, Centre of Biological Engineering, University of Minho, Braga, Portugal; 5 Laboratory of Medical Investigation (LIM14), Faculty of Medicine of São Paulo University, São Paulo, Brazil; 6 Molecular Oncology Research Center, Barretos Cancer Hospital, Barretos, Brazil; Fondation Raoul Follereau, FRANCE

## Abstract

Buruli Ulcer (BU) is a necrotizing skin disease caused by *Mycobacterium ulcerans* infection. BU is characterized by a wide range of clinical forms, including non-ulcerative cutaneous lesions that can evolve into severe ulcers if left untreated. Nevertheless, spontaneous healing has been reported to occur, although knowledge on this process is scarce both in naturally infected humans and experimental models of infection. Animal models are useful since they mimic different spectrums of human BU disease and have the potential to elucidate the pathogenic/protective pathway(s) involved in disease/healing. In this time-lapsed study, we characterized the guinea pig, an animal model of resistance to *M*. *ulcerans*, focusing on the macroscopic, microbiological and histological evolution throughout the entire experimental infectious process. Subcutaneous infection of guinea pigs with a virulent strain of *M*. *ulcerans* led to early localized swelling, which evolved into small well defined ulcers. These macroscopic observations correlated with the presence of necrosis, acute inflammatory infiltrate and an abundant bacterial load. By the end of the infectious process when ulcerative lesions healed, *M*. *ulcerans* viability decreased and the subcutaneous tissue organization returned to its normal state after a process of continuous healing characterized by tissue granulation and reepethelialization. In conclusion, we show that the experimental *M*. *ulcerans* infection of the guinea pig mimics the process of spontaneous healing described in BU patients, displaying the potential to uncover correlates of protection against BU, which can ultimately contribute to the development of new prophylactic and therapeutic strategies.

## Author Summary

Buruli Ulcer (BU) is a devastating skin disease caused by *Mycobacterium ulcerans*. BU usually starts off as a non-ulcerative lesion, but if lesions are left untreated they can evolve into ulcers or may even affect the bone. Nevertheless, spontaneous healing of active lesions has been reported in some patients, although little is known about this process. In this study, the authors performed a time-lapsed study on a resistant animal model of *M*. *ulcerans* infection–the guinea pig. Subcutaneous infection of the guinea pig led to the development of ulcerative lesions that eventually healed over the course of infection and, interestingly, this healing was associated with a decrease in *M*. *ulcerans* viability and with an ongoing reparative process of the infected tissue. Given that similar observations have been made in BU patients that spontaneously resolved *M*. *ulcerans* infection, the guinea pig model has the potential to disclose the protective immune mechanisms underlying resistance to BU.

## Introduction

Buruli Ulcer (BU) is a necrotizing skin disease caused by *Mycobacterium ulcerans*. *M*. *ulcerans* pathogenesis is mediated by mycolactone, a potent polyketide-derived macrolide that triggers apoptotic cell death [1,2]. Initial pre-ulcerative BU lesions are indolent with no systemic symptoms, which often results in a delay in health-care seeking. If lesions are left untreated, they can evolve into severe extensive ulcers with characteristic undermined edges and a necrotic sloughed center [3]. In the most extreme cases, *M*. *ulcerans* may invade the bone [4,5].

Spontaneous healing of active lesions is thought to be frequent [6–8], although the rate at which this occurs has not been specifically addressed. Therefore, all forms of BU are subjected to the standard first-line treatment, consisting of daily administration of streptomycin and rifampicin for an 8-12-week period [9]. Additional surgical debridement and skin grafting may also be required in the case of extensive lesions. The healing process of severe lesions, whether spontaneous or antibiotic-induced, often results in atrophic scarring, contractures, and severe functional disabilities, ultimately having a negative impact on socioeconomical development [10]. This is why prevention, early detection and treatment have been a priority for the control of BU. Therefore, a better knowledge on the nature of protective responses against *M*. *ulcerans* could improve patient management. In that sense, animal models of infection have been extensively used, not only to characterize *M*. *ulcerans* disease progression [11], but also to dissect immunological pathways [12–16] and to evaluate the efficiency of antibiotic regimens [17–21] or vaccine candidates [22–27].

The most common animal model used for the study of *M*. *ulcerans* infection has been the mouse, in which experimental infection was shown to be progressive, leading to an exponential increase in bacterial burdens, a continuous destruction of infiltrating cells, expansion of necrosis into healthy tissue and ulceration of the epidermis, similar to what is observed in active, progressive human BU [12,28]. The guinea pig model has also been used for the study of *M*. *ulcerans* infection [1,11,29–31]; however, definitive experimentation on the nature of disease progression in this animal model has been limited. Therefore, in this study, we proposed to perform a detailed characterization of the progression of *M*. *ulcerans* infection in the guinea pig model, focusing on macroscopic, microbiological and histological evolution.

## Results

### Spontaneous healing of ulcerative lesions and clearance of *M*. *ulcerans* in infected guinea pigs

The mouse has been the animal model of choice to characterize progressive *M*. *ulcerans* disease [11], to dissect immunopathologic pathways [12–16] and to evaluate the efficiency of antibiotic regimens [17–21] or vaccine candidates [22–27]. Although less explored, the guinea pig model has also been used to study *M*. *ulcerans* infection, with published reports focusing mainly on the evolution of the macroscopic features and on the histopathology of early time points [11,29–31]. In this study, we decided to explore this overlooked animal model, by performing a time-lapsed study of the macroscopic, histological, and microbiological evolution of *M*. *ulcerans* infection in the ear or the back of the guinea pig.

**Fig 1:**
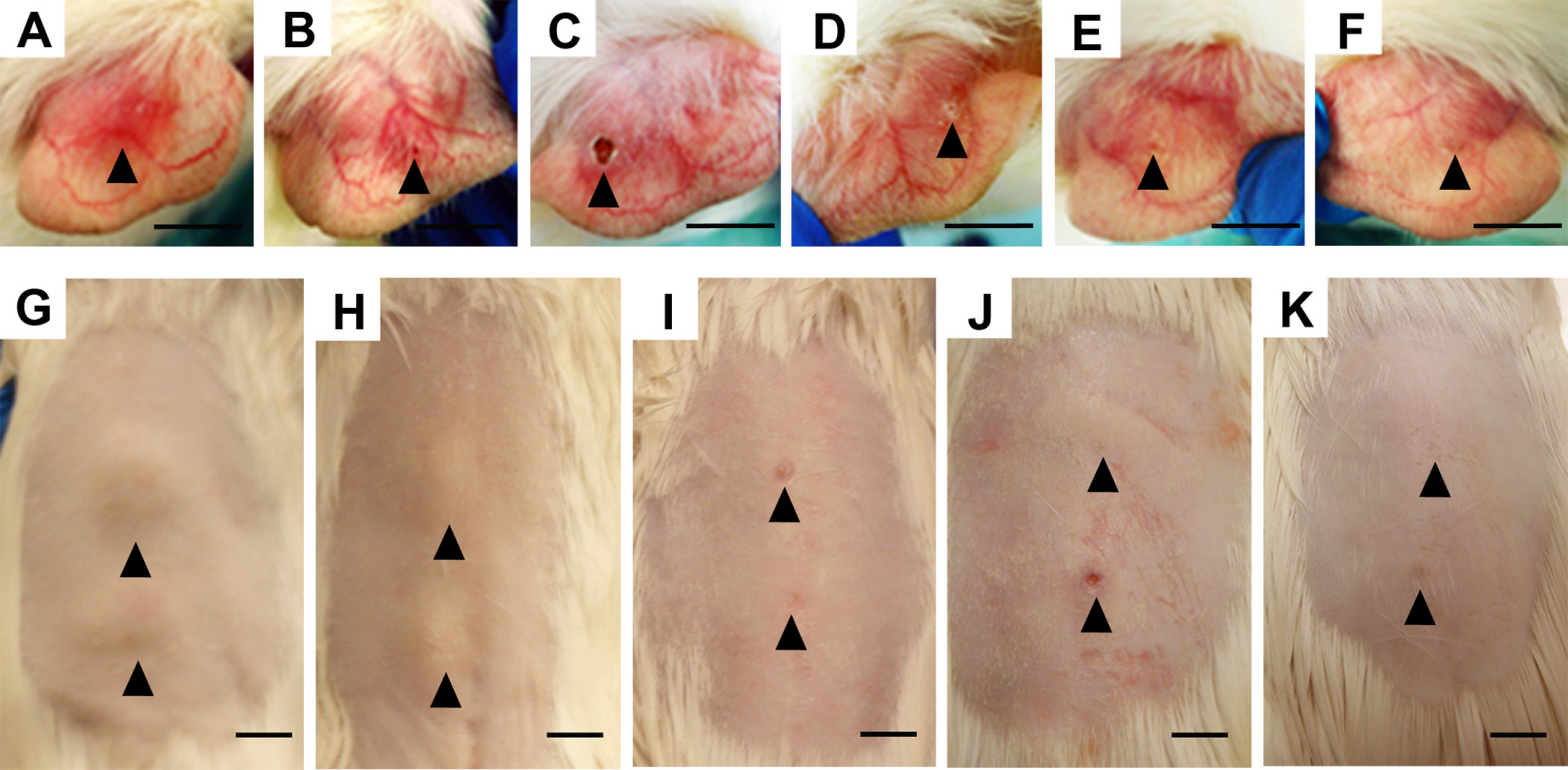
Macroscopic progression of *M*. *ulcerans* infection in the guinea pig model. Guinea pigs were subcutaneously infected in the ear (A-F) or in the back (G-K) with approximately 4log_10_ CFU of *M*. *ulcerans* strain 98–912. The macroscopic progression of the lesions at the site of infection was photographed over the course of experimental infection in the ear: (A) 4days; (B) 8days; (C) 14days; (D) 18days; (E) 26days; (F) 35days; or in the back (G) 8days; (H) 12days; (I) 16days; (J) 21days; (K) 35days. Arrowheads represent the site of subcutaneous *M*. *ulcerans* infection. One experiment representative of three total experiments is shown. Scale: 1cm.

**Fig 2:**
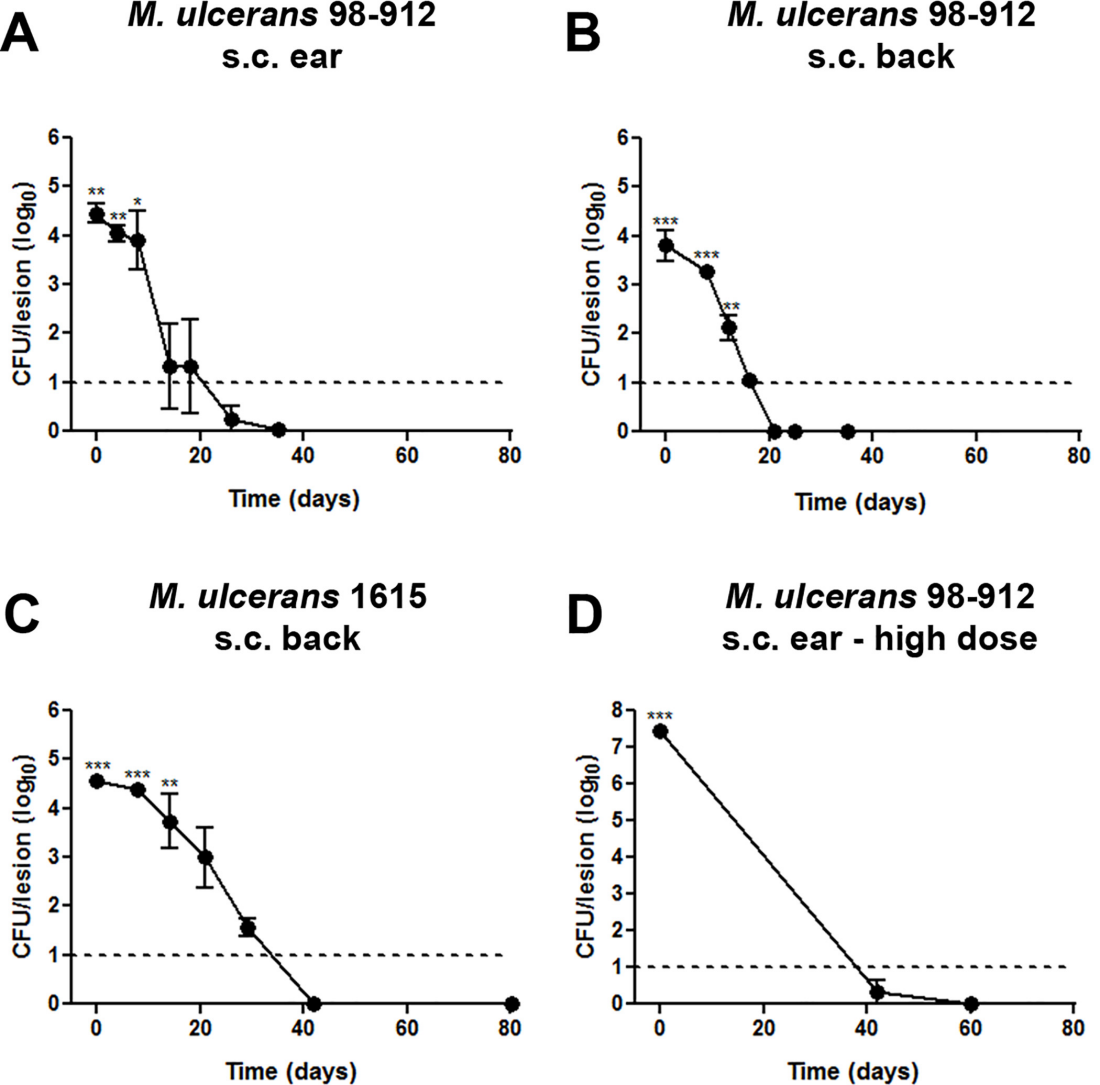
Evolution of bacterial viability of *M*. *ulcerans* in the guinea-pig model. Guinea pigs were subcutaneously infected (A) in the ear with *M*. *ulcerans* strain 98–912; (B) in the back with *M*. *ulcerans* strain 98–912; (C) in the back with *M*. *ulcerans* strain 1615 or (D) in the ear with a high initial infectious dose of *M*. *ulcerans* strain 98–912. At different time points post-infection, infected tissue was harvested and viable bacterial counts were performed. Dashed line represents the detection limit. Data points represent the mean ± SEM (n = 4–5) for each time point. One experiment representative of three total experiments is shown. Statistical significance was calculated with One way ANOVA, post-hoc Tukey test. Presented p-values correspond to the comparison of all time points with the last time point for which the bacterial load is above the dashed line (** p<0.01; ***p<0.001).

Initially, subcutaneous infection with *M*. *ulcerans* 98–912 in the ear or back of guinea pigs led to early focal indurated erythema (Fig 1A, 1B, 1G and 1H), reminiscent of a nodule-like structure. During the course of infection, these lesions progressed to small ulcers with overlying scabs (Fig 1B, 1C, 1I and 1J). Between days 18–26, scabs detached revealing a healed and reepithelialized lesion, with occasional scarring (Fig 1D–1F and 1K). An important observation was that these macroscopic alterations were accompanied by bacterial clearance, regardless the site of infection (Fig 2A and 2B), the infecting strain (*M*. *ulcerans* 98–912—Fig 2B or *M*. *ulcerans* 1615—Fig 2C) or the infectious dose (4.5log_10_ CFU—Fig 2A or 7.4log_10_ CFU—Fig 2D).

### Histological analysis of the healing process in *M*. *ulcerans*–infected tissue of guinea pigs

**Fig 3:**
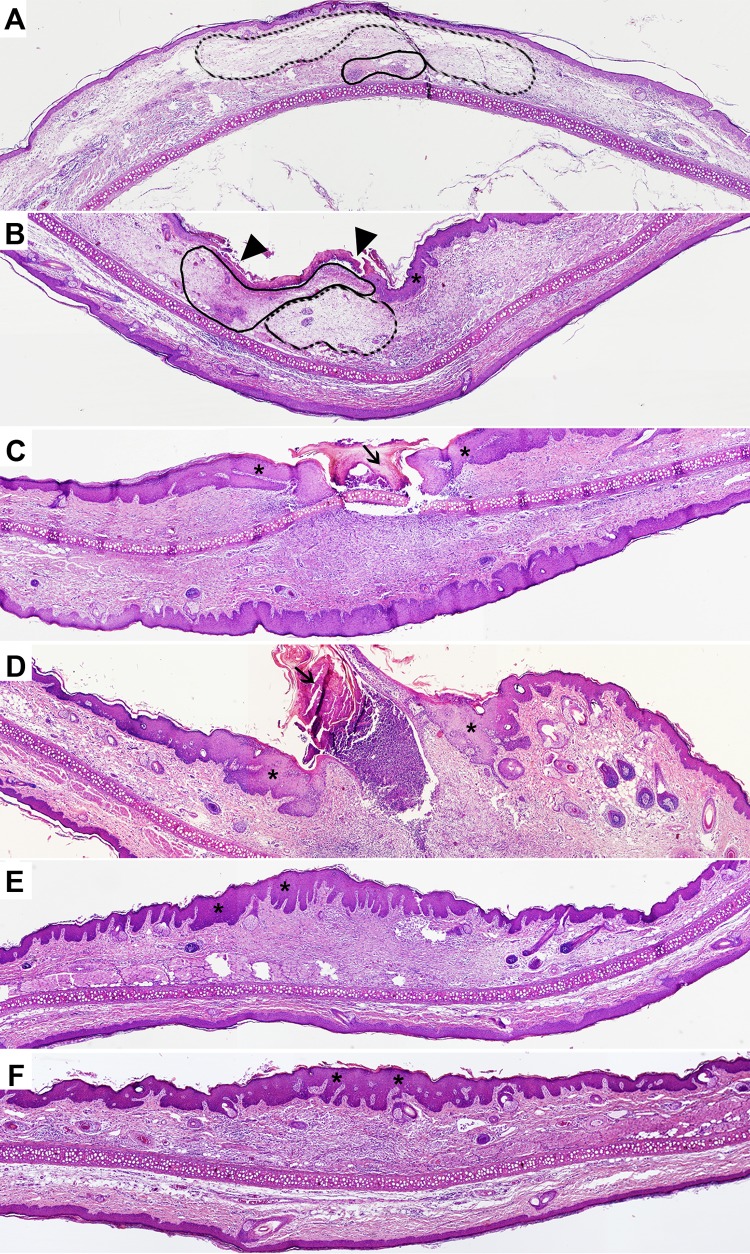
Histological overview of *M*. *ulcerans* infected skin in the guinea pig model. Guinea pigs were subcutaneously infected in the ear with 4.5 log_10_ CFU of *M*. *ulcerans* strain 98–912. Histological sections of ears collected at different time points were stained with HE. Magnification of images 4x. At day 4 (A) and day 8 (B), edema (dotted line) and necrosis (full line), associated with a surrounding inflammatory, infiltrate were the predominant features. By day 8, necrosis expanded throughout the tissue, resulting in epidermal ulceration (triangles). Day 14 (C) and day 18 (D), were characterized by the presence of an eschar (arrows) over the wounded surface and epidermal hyperplasia (asterisks). By day 26 (E) and day 35 (F), complete repethilialization was evident, as well as epidermal hyperplasia and elongation of rete ridges (asterisks).

**Fig 4:**
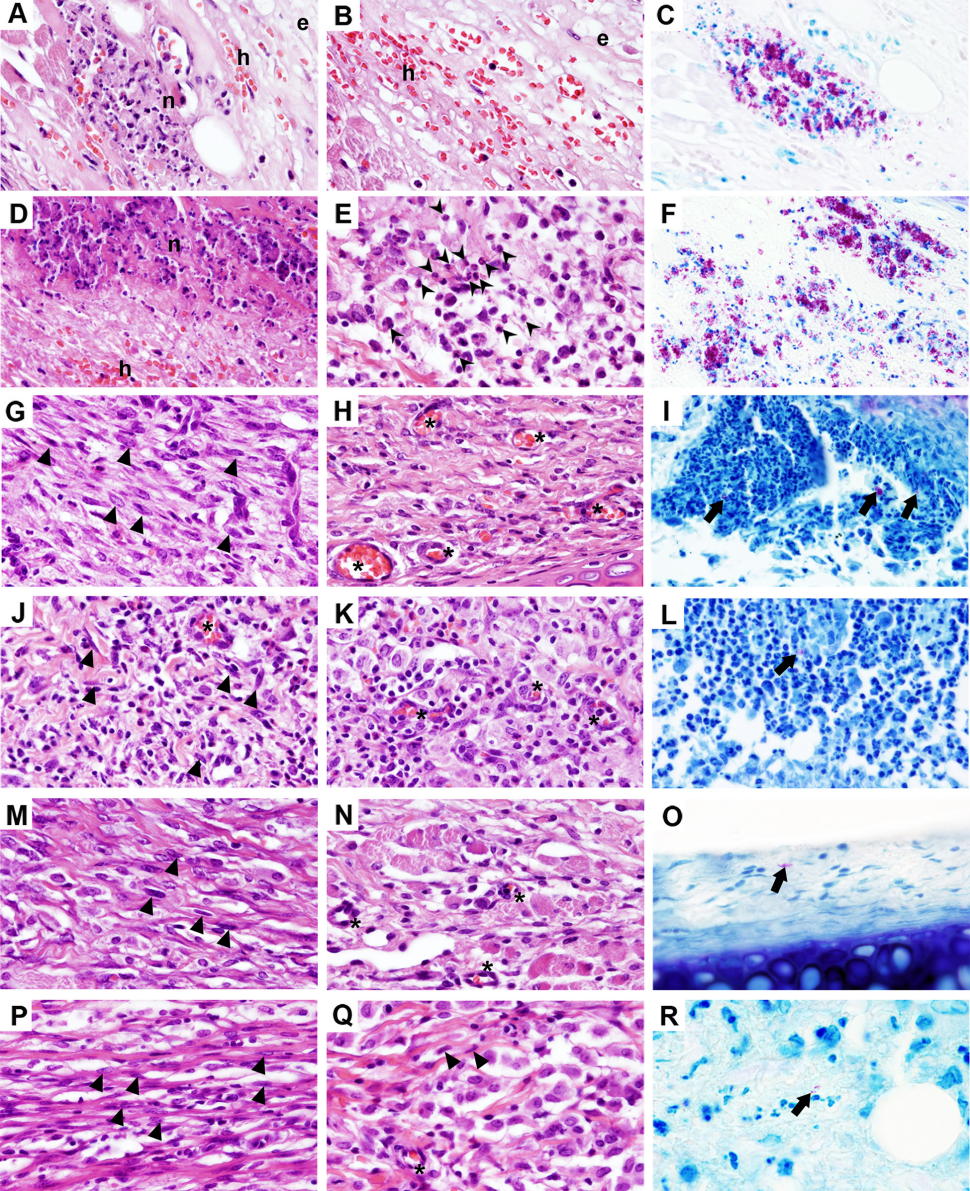
Histological details of *M*. *ulcerans* infected skin in the guinea pig model. Guinea pigs were subcutaneously infected in the ear with 4.5 log_10_ CFU of *M*. *ulcerans* strain 98–912. Histological sections of ears collected at different time points were stained with HE (A-B, D-E, G-H, J-K, M-N, P-Q) or ZN (C, F, I, L, O, R). Magnification of images 60x. At day 4 (A-C) and day 8 (D-F), necrotic foci (n), edema (e), hemorrhage (h), and an acute inflammatory infiltrate (arrowheads) were the main features of the infected tissue. By day 14 (G-I) and day 18 (J-L), initiation of wound healing was evident by the presence of granulation tissue, characterized by a fibroblastic population (triangles) and neovascularization (asterisks). At day 26 (M-O) and day 35 (P-R), fibroblasts (traingles) and newly formed vessel (asterisks) were still present and there was an increased deposition of elongated pink collagen fibers. The bacterial load (C, F, I, L, O, R) decreased over the course of infection.

In line with the macroscopic observations described above, histological analysis of the infected skin of the guinea pig showed signs of a dynamic infectious process culminating with a tissue reparation and bacterial clearance (Figs 3 and 4).

During the first week of infection, the underlying dermis was edematous (Figs 3A and 4B) and focally necrotic (Figs 3A, 3B and 4A–4F), with diffuse infiltration of neutrophils and histiocytes (Fig 4E). Bacteria were mainly concentrated in clumps within the necrotic foci, but could also be found co-localized with phagocytic cells (Fig 4C and 4F). These necrotic areas expanded progressively during the first week of infection, resulting both in an increase in cells with apoptotic morphology (Fig 4D) and in an initial epidermal destruction (Fig 4B). However, by day 14 post-infection, signs of active tissue healing were evidenced by a sloughing of the mycobacteria-rich necrotic tissue (Fig 3C and 3D), accumulation of fibroblasts in the subcutaneous tissue (Fig 4G and 4J), intense neovascularization (Fig 4H and 4K) and granulation tissue that gradually replaced the early acute cellular infiltrate (Fig 4G, 4H, 4J and 4K). Analysis of the infected tissue also indicated an ongoing process of skin reepithelialization, evidenced by the formation of an eschar composed of degenerating epidermis and collagen over the ulcerated wound, and also by the thickening of the adjacent epidermal layer (Fig 3C and 3D). Interestingly, few bacilli were scattered throughout the granulation tissue, being mainly found nearby the wound surface (Fig 4I and 4L). By the end of the experimental period of infection (day 26–35), reepithelialization was complete (Fig 3E and 3F), although fibroplasia (increased deposition of collagen fibers) (Fig 4M, 4N, 4P and 4Q) and epidermal hyperplasia (elongation of rete ridges) (Fig 3E and 3F) were still evident. In accordance with the CFU counts shown in Fig 2, rare or no bacilli could be observed in the infected tissue by day 26 post-infection and until the end of the experimental period (Fig 4O and 4R).

## Discussion

*M*. *ulcerans* is a human pathogen, which has recently been shown to naturally infect other mammals in Australia [32–35]. Regarding experimental models of infection, early studies have established the capacity of *M*. *ulcerans* to infect a variety of mammals, namely mice and guinea pigs [11,29,30].

It has been well demonstrated that experimental *M*. *ulcerans* infection in the murine model mimics the main features of active, progressive BU [36] (S1 Fig). Indeed, infection with virulent *M*. *ulcerans* strains induces a persistent acute inflammatory response throughout infection [12]; however, mycolactone destroys the recruited inflammatory infiltrates generating necrotic acellular areas with extracellular bacilli released by the lysis of infected phagocytes [12,28]. These necrotic areas gradually expand, through the progressive invasion of healthy tissues, eventually resulting in ulceration of the epidermis [12,28].

The guinea pig model, on the other hand, has been occasionally used in BU research, namely to characterize the pathogenicity of *M*. *ulcerans* and its toxin [1,11,29–31]. It has been described that following intradermal infection guinea pigs developed initial localized swelling followed by the appearance of ulcerative lesions. Histologically, these lesions were characterized by necrosis, clumps of AFB and diffuse infiltration of polymorphonuclear cells and histiocytes [29,30]. Interestingly, these ulcerative lesions ultimately healed, although further analysis of the healing phase was not explored [29,30].

In the present study, we confirm the previous observations in the guinea pig model and further these findings with a time-lapsed histological and microbiological analysis of *M*. *ulcerans* infected tissues. At early time points, infected guinea pig tissue showed focal necrotic areas harbouring intense bacterial loads, compromised epidermal layers, and recruitment of neutrophils and mononuclear cells to the infectious foci. This acute inflammatory profile was gradually replaced by granulation tissue and it is during this transition phase that we observe a decrease in *M*. *ulcerans* viability. By the end of the infectious process, we observed complete reepithelialization of the wound surface and scarce/absent bacilli in the tissue. This study contributes to raise relevant questions that should be addressed in future studies, such as whether the immune response plays a role in the control of *M*. *ulcerans* proliferation; whether lesions are completely sterilized or if *M*. *ulcerans* remains latent in the tissue; and whether cells from the guinea pig or the mouse present a differential susceptibility to mycolactone.

Similar observations have been made in BU patients that spontaneously resolved *M*. *ulcerans* infection before developing extensive destructive lesions [6–8]. Connor and Lunn were the first to propose three histopathological stages in human *M*. *ulcerans* infection: the stage of necrosis, the organizing stage, and the healing stage [6], all of which were identified in the guinea pig tissue during *M*. *ulcerans* experimental infection. Additionally, Revill et al. reported the spontaneous healing of small BU lesions in 29% of the clinically diagnosed patients that were receiving placebo during a randomized study of clofazamine therapy [7]. More recently, Gordon et al. reported a case in which a patient became IS*2404* PCR, ZN and culture negative, one month after laboratory confirmation of initial BU diagnosis [8]. This decrease in bacterial viability also coincided with the presence of mixed inflammatory cell infiltration and granulation tissue [8]. Taking into consideration these similarities, studies on the guinea pig model have the potential to disclose the protective mechanisms underlying resistance to BU in humans.

More recently, other animal models of *M*. *ulcerans* infection have been proposed to better understand the early host-pathogen interactions and the pathogenesis of BU, namely the monkey [37] and pig models [38]. The cynomolgus monkey developed papules that progressed to ulcers with undermined borders after *M*. *ulcerans* infection. Histological analysis of biopsies from ulcer edges showed necrosis, robust inflammatory infiltrates, granulomatous-like responses, mild edema, and extracellular acid-fast bacilli [37]. Similarly to the guinea pig, ulcers eventually healed spontaneously with no signs of systemic infection [37]. On the other hand, in the porcine model, infection with *M*. *ulcerans* leads to the development of nodular lesions that subsequently progress to ulcers [38], similar to what we describe here for the guinea pig. However, in the porcine model, only low doses of *M*. *ulcerans* infection lead to spontaneous healing of ulcerative lesions, while high doses resulted in progressive infection during the experimental period [38]. Among these animal models of resistance, the guinea pig presents several advantages, namely smaller size, lower cost and maintenance, making it a more accessible model to study mechanisms of protection against *M*. *ulcerans* infection.

In conclusion, both the mouse and the guinea pig are useful experimental models since they mimic different spectrums of human *M*. *ulcerans* disease, therefore contributing to elucidate the pathogenic/protective pathway(s) involved in disease-progression/healing, respectively. Here we show that further studies on the cellular and molecular components of the protective response in the guinea pig model have the potential to uncover correlates of protection against BU.

## Methods

### Bacteria

*M*. *ulcerans* 98–912 (ITM collection, Antwerp, Belgium) is a mycolactone D producing strain isolated in China from an ulcerative lesion [39]. *M*. *ulcerans* 1615 is a mycolactones A/B producing strain isolated in Malaysia from an ulcerative case. The isolates were grown on Middlebrook 7H9 medium (Becton, Dickinson and Company) with agar at 32°C for approximately 6–8 weeks. For the preparation of the inoculum, *M*. *ulcerans* was recovered, diluted in phosphate-buffered saline (PBS) and vortexed using glass beads.

### *M*. *ulcerans* experimental infection

Female Hartley guinea-pigs and Balb/c mice were obtained from Charles River (Barcelona, Spain) and were housed in specific pathogen free conditions with food and water *ad libitum*. Before experimental infection, guinea pigs were anesthetized with 40mg/kg of ketamine and 0.5mg/kg of medetomidine via intraperitoneal (i.p.) injection. Guinea pigs were infected subcutaneously (s.c.) in the dorsal area (after the removal of the fur) or the ear with 100μl of *M*. *ulcerans* strain 98–912 or 1615. After infection, anesthesia was reverted with 1mg/kg of atipamezol via i.p. injection. At different time points post-infection, guinea pigs were sacrificed by i.p. injection of 150mg/kg of pentobarbital. In parallel, mice were anesthetized with 75mg/kg ketamine and 1mg/kg medetomidine administered via i.p. and were then s.c. infected in the ear with 10μl of *M*. *ulcerans* inoculum. Anesthesia was reverted with 1mg/kg of atipamezol via i.p. injection. Mice were sacrificed by asphyxiation with increasing concentrations of CO_2_. For each experiment, the susceptible mouse model was used to confirm of the virulence of the *M*. *ulcerans* strain.

### Bacterial load determination

Infected tissue was excised, homogenized and diluted in PBS. Suspensions were decontaminated with hydrochloric acid (HCl) 1M containing 0,001% phenol red solution for 15min at room temperature, followed by neutralization with sodium hydroxide (NaOH) 1M. Suspensions were centrifuged and the pellet resuspended in PBS. Serial dilutions of the suspension were plated on nutrient 7H9 + 1,5% agar. Bacterial colony formation was counted after 6–8 weeks of incubation at 32°C. Following ASTM guidelines, the reported value for the limit of detection for microbiological purposes should be “< the dilution value” if no colonies are recovered; therefore the limit of detection for our experiments is 1log_10_CFU/ml. All countable colonies, even those below the countable range, were counted and reported as an estimated count.

### Histological studies

Infected tissue was fixed in buffered formalin and embedded in paraffin. Light-microscopy studies were performed on tissue sections stained with hematoxylin and eosin (HE) or Ziehl-Neelsen (ZN). Images were obtained with an Olympus BX61 microscope.

### Statistical analysis

Differences between the means of experimental groups were analyzed with the One Way Anova-test post-hoc Tukey. Differences with a p-value of ≤0.05 were considered significant.

### Ethics statement

This study was approved by the Portuguese National Authority for animal experimentation *Direcção Geral de Alimentação e Veterinária*. Animals were kept and handled in accordance with the guidelines for the care and handling of laboratory animals in the European Union Directive 86/609/EEC.

## Supporting Information

S1 Fig Macroscopic progression of *M*. *ulcerans* infection in the mouse ear model. Balb/c mice were subcutaneously infected in the ear with approximately 6log_10_ CFU of *M*. *ulcerans* strain 98–912. The macroscopic progression of the lesions at the site of infection were photographed over the course of experimental infection: (A) 3 days; (B) 14 days; (C) 27 days; (D) 55 days; (E) 70 days; (F) 105 days. One experiment representative of two total experiments is shown. Scale: 1cm.
(TIF)
