# Supplementary figures and images for: Spontaneous Healing of Mycobacterium ulcerans Lesions in the Guinea Pig Model
Source: PLoS Negl Trop Dis. 2015 Dec 1;9(12):e0004265. doi: 10.1371/journal.pntd.0004265 (PMC4666642; doi:10.1371/journal.pntd.0004265)

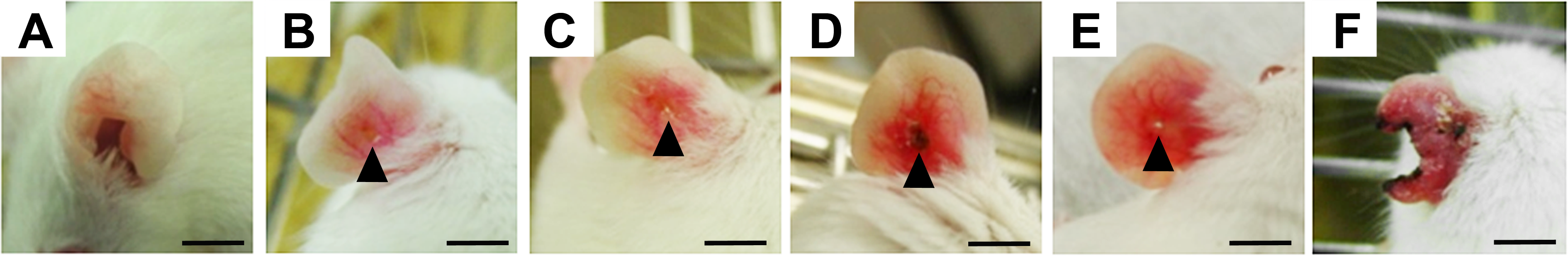

Supplement: S1 Fig — Balb/c mice were subcutaneously infected in the ear with approximately 6log10 CFU of M. ulcerans strain 98–912. The macroscopic progression of the lesions at the site of infection were photographed over the course of experimental infection: (A) 3 days; (B) 14 days; (C) 27 days; (D) 55 days; (E) 70 days; (F) 105 days. One experiment representative of two total experiments is shown. Scale: 1cm. (TIF) [file pntd.0004265.s001.tif]
